# Potentiated GABAergic neuronal activities in the basolateral amygdala alleviate stress‐induced depressive behaviors

**DOI:** 10.1111/cns.14422

**Published:** 2023-09-16

**Authors:** Muhammad Asim, Huajie Wang, Xi Chen, Jufang He

**Affiliations:** ^1^ Department of Neuroscience City University of Hong Kong Kowloon Tong People's Republic of China; ^2^ Department of Biomedical Science City University of Hong Kong Kowloon Tong People's Republic of China; ^3^ City University of Hong Kong Shenzhen Research Institute Shenzhen People's Republic of China

**Keywords:** basolateral amygdala, CAMKII, depression, GABA

## Abstract

**Aims:**

Major depressive disorder is a severe psychiatric disorder that afflicts ~17% of the world population. Neuroimaging investigations of depressed patients have consistently reported the dysfunction of the basolateral amygdala in the pathophysiology of depression. However, how the BLA and related circuits are implicated in the pathogenesis of depression is poorly understood.

**Methods:**

Here, we combined fiber photometry, immediate early gene expression (c‐fos), optogenetics, chemogenetics, behavioral analysis, and viral tracing techniques to provide multiple lines of evidence of how the BLA neurons mediate depressive‐like behavior.

**Results:**

We demonstrated that the aversive stimuli elevated the neuronal activity of the excitatory BLA neurons (BLA^CAMKII^ neurons). Optogenetic activation of CAMKII neurons facilitates the induction of depressive‐like behavior while inhibition of these neurons alleviates the depressive‐like behavior. Next, we found that the chemogenetic inhibition of GABAergic neurons in the BLA (BLA^GABA^) increased the firing frequency of CAMKII neurons and mediates the depressive‐like phenotypes. Finally, through fiber photometry recording and chemogenetic manipulation, we proved that the activation of BLA^GABA^ neurons inhibits BLA^CAMKII^ neuronal activity and alleviates depressive‐like behavior in the mice.

**Conclusion:**

Thus, through evaluating BLA^GABA^ and BLA^CAMKII^ neurons by distinct interaction, the BLA regulates depressive‐like behavior.

## Graphical abstract

Under normal conditions, BLA neurons are in a resting state and maintained the balance in inhibition/excitation. However, stress leads to the overactivity of BLA^CAMKII^ neurons and mediates the depressive‐like phenotypes. Enhancing the inhibition in the BLA either through chemogenetic activation of BLA^GABA^ neurons or direct inhibition of BLA^CAMKII^ neurons alleviates depressive‐like behaviors.

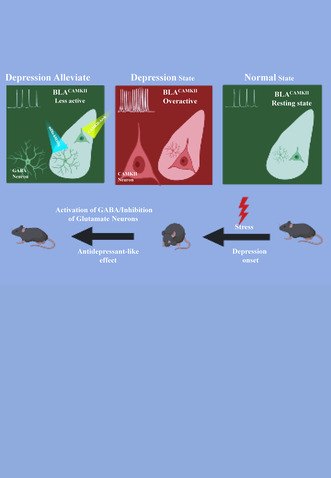

## 1 INTRODUCTION

Depression is a major cause of disability globally and a key contributor to the overall global burden of diseases with a lifetime prevalence (~17%).^1^, ^2^ Chronic stress is considered a major risk factor for depression, among the convoluted etiologies such as genetic vulnerability, neuroendocrine alteration, and aging.^3^ Stress is the foremost cause of synaptic dysfunction, which can be a potential therapeutic target for stress‐related mood disorders, including depression.^4^, ^5^ Stress leads to neural plasticity changes in the brain's valence‐coding systems that are strongly associated with depression.^6^, ^7^, ^8^, ^9^ The basolateral amygdala (BLA) is regarded as prominent among numerous valence‐coding brain regions. The BLA plays a crucial role in orienting and processing threats.^10^ BLA reactivity may emphasize mood disorders, heightened anxiety, biased emotional processing, and preferential representation of negative information typical of depression.^11^ Besides negative emotional responses, BLA has also been implicated in safety signal processing and reward behaviors.^12^, ^13^, ^14^, ^15^

The BLA consists of a majority (80%–85%) of spiny glutamatergic neurons and a minority (up to 20%) of GABAergic neurons.^16^, ^17^ The BLA^CAMKII^ projection neurons downstream target the medial prefrontal cortex and nucleus accumbens and could hijack their activity under stress conditions.^17^, ^18^, ^19^ Hyperactivity of BLA during major depression is one of the consistent findings in clinical neuroscience.^20^, ^21^ Therefore, enhancing the inhibition in the BLA could be a potential treatment for depression. We recently found that the high‐frequency activation of GABAergic neurons in the auditory cortex enhances inhibition and reduces glutamatergic neuronal activity in response to auditory stimuli.^22^ Regardless of proof from human experiments accusing changes in volume,^23^ metabolism,^24^, ^25^ and valence response^26^ of the BLA in depressed patients, how BLA individual neurons (BLA^GABA^ and BLA^CAMKII^) are engaged in the pathogenesis of depression remains largely unexplored.

Here, we adopted fiber photometry to investigate how aversive stimuli affect the BLA neuronal activity. Furthermore, we investigated how optogenetic stimulation affects real‐time behavior. Next, we explored whether the inhibitory GABAergic neurons in the BLA present major functional input to BLA^CAMKII^ neurons at the neural activity by using fiber photometry and behavioral level using chemogenetics and optogenetic methods. Finally, we used retrograde labeling to find the input sources to BLA.

## 2 MATERIALS AND METHODS

### 2.1 Animals

All experiments were certified by the Animal Advisory Committee at the City University of Hong Kong Guidelines for the Care and Use of Laboratory Animals. Male C57BL/6 J (8–12 weeks old) of normal appearance and weight were used for all behavioral tests, immunohistochemistry, fiber photometry, optogenetics, and chemogenetics experiments. Ai14 mice were used for retrograde labeling. All male mice were socially housed, five per cage, and kept under typical housing environments on corn cob litter in a temperature (23 ± 1°C) animal room on a 12 h light/dark cycle (lights were on from 8:00 to 20:00 every day) with food and water ad libitum until surgery. All the behavioral experiments were performed in the night‐time (Table S1).

### 2.2 Viruses

The viruses AAV9‐CAMKII‐GCAMP6s (viral: 2.50 × 10^13^), AAV9‐Dlx‐hM4Di‐dtomato‐Fishell‐5 (viral: 3.30 × 10^13^), and Retro‐AAV‐hSyn‐Cre (viral: 1.8 × 10^13^) were purchased from Addgene. The virus AA9‐mDlx‐DIO‐ChrimsonR‐mcherry (viral: 5.09 × 10^12^), AAV9‐mDlx‐DIO‐mCherry (viral: 2.50 × 10^12^), AAV9‐mDlx‐DIO‐hM3Dq‐EGFP (viral: 5.54 × 10^12^), AAV9‐mDlx‐Cre (viral: 2.45 × 10^12^), AAV9‐mCamKII‐Cre (viral: 5.21 × 10^12^), and AAV9‐EF1a‐DIO‐EYFP (viral: 5.24 × 10^12^) were purchased from BrainVTA company. The virus AAV9‐mCamKII‐DIO‐ChrimsonR‐mCherry (Viral: 1.26 × 10^13^), AAV9‐hSyn‐DIO‐eArchT3.0‐EGFP (viral: 1.02 × 10^13^), and AAV9‐hSyn‐DIO‐EGFP (viral: 2.13 × 10^13^) were bought from Shanghai Taitool Bioscience, China. Viruses with 10^13^ particles were used after 2 times dilution (Table S1).

### 2.3 Virus injection and optical fiber implantation

Mice were anesthetized with sodium pentobarbital (50 mg/kg, i.p. injection) for bilateral stereotaxic injection of viruses into the BLA (AP: −1.60; ML: ±3.40; DV: −4.00; mm AP and ML are corresponding to bregma and DV from dura matter; AP, ML, and DV refer to the anteroposterior, mediolateral, and dorsoventral distance from the bregma, respectively). The coordinates were determined from the bregma corresponding to the mouse brain atlas. We infused 300 nL of the virus into each side at a rate of 50 nL/min. After each injection, the needle was kept in place for another 10 min and then gradually withdrawn.

To record the fiber photometry GcaMP signal mice were implanted with an optical fiber cannula (length 6 mm, NA 0.37) held in a ceramic ferrule (Inper company) over the BLA (AP: −1.60; ML: ±3.40; DV: −3.85; mm AP and ML are corresponding to bregma and DV from dura matter. For optogenetic stimulation, mice were implanted with a bilaterally optical fiber cannula (length 6 mm, NA 0.37) held in a ceramic ferrule (Inper company) over the BLA (AP: −1.60; ML: ±3.40; DV: −3.70; mm AP and ML are corresponding to bregma and DV from dura matter.

### 2.4 Fiber photometry‐based calcium measurements

Mice were attached with a fiber patch cord and allowed to habituate for a minimum of 10 min prior to each testing session. To record fluorescence signals, the fiber‐photometry system (Doric Lenses) used two continuous sinusoidally modulated LEDs (DC4100, ThorLabs) at 473 nm (220 Hz) and 405 nm (330 Hz) as light sources to excite GCaMP6s and an isosbestic autofluorescence signal, respectively. Both lights were connected to a large‐core (200 μm), high‐NA (0.37) optical fiber patch cord, which was coupled with a corresponding brain implant in each mouse. The light intensity at the interface between the fiber tip was set to 15 μW. GCaMP6s and autofluorescence signals were gathered by the same fiber and driven onto two separate photoreceivers (2151, Newport Corporation). An RZ5P acquisition system (Tucker‐Davis Technologies; TDT), fitted out with a real‐time signal processor, operated the LEDs and also autonomously demodulated the fluorescence brightness due to 473‐nm and 405‐nm excitations. Behavioral activities were recorded by the same system via TTL input.

The acute tail suspension protocol for fiber photometry was adopted from a previous study.^27^ Briefly, animals were connected to an optical patch cord and permitted to habituate for 10 min. After that, animals were suspended with their tails by a single experimenter for a total of three trials, each lasting 30 s every 2 min. Changes in baseline fluorescence during the acute tail suspension test (TST) were calculated by evaluating the 30 s of recording data during tail suspension to the 5 s before the onset of the TST. For the social interaction task, mice were permitted to habituate for 10 min with an optical patch cord. After that, animals were placed into the social interaction chamber by a single experimenter for a total of 5 min. For the social interaction test (SIT), stimulated changes in fluorescence were calculated by comparing the recording data 5 s before social interaction and 10 s after mice entered the CD‐1 aggressor mouse interaction zone. In a separate group of animals, foot shock‐induced neural activity was analyzed by comparing the 5 s before foot shock and 10 s after foot shock. Data were analyzed by pMAT an open‐source software suite for the analysis of fiber photometry data.^28^ The resulting fitted 405‐nm signal was then used to normalize the 473‐nm signal as follows: ΔF/F0 = (473‐nm signal − fitted 405‐nm signal)/fitted 405‐nm signal.

### 2.5 Optogenetic alleviation of depressive‐like behavior

Mice injected with either eArchT3.0 or EYFP (control) in the BLA of C57 mice. For optogenetic inhibition of BLA^CAMKII^ neurons mice were attached to an optical patch cord and placed on opposite sides of CD‐1 aggressor mice with the divider. Mice received yellow light inhibition for 4 s on and 1 s off for 10 min for three consecutive days post chronic social defeat stress.

### 2.6 Chemogenetic enhancement of depressive‐like behavior

Mice injected with either hM4Di or mcherry (control) virus in the BLA of C57 mice. For chemogenetic facilitation of depressive‐like behavior with subthreshold social defeat stress. Mice received 5 mg/kg CNO immediately after the first trial of two trials of social defeat and were kept on opposite sides of the CD‐1 aggressor for 15 min before the second trial of physical defeat. After the second trial mice were kept in their home cage and behavior test were conducted the following days to evaluate depressive‐like behavior.

### 2.7 Immunohistochemistry

Mice were anesthetized by an overdose of pentobarbital sodium 60 min after sensory stimuli and transcardially perfused with 30 mL PBS, followed by 30 mL of 4% paraformaldehyde (PFA) in PBS. Brains were removed and fixed with 4% PFA overnight. Coronal sections 50 μm in thickness were cut using a vibratome. Brain sections were first washed with PBS and then blocked with 5% goat serum in PBST (0.3% Triton X‐100 in PBS) for 2 h. Then slices were incubated with primary antibody anti‐c‐fos rabbit (ab190289) with a concentration of (1:1000) for 24 h at 4°C. Slices were washed 3 times with PBS and incubated with secondary antibodies either Alexa fluor 488 (1:500) or Alexa fluor 647 (1:500) at room temperature for 2 h, followed by washing 3 times with PBS and then incubated with DAPI (1:5000) for 5 min and washed again with PBS for 3 times. Slices were mounted on slides and images were taken with a Nikon confocal microscope. Data were analyzed with Image J software (Table S1).

### 2.8 Behavior assays

#### 2.8.1 Real‐Time Place Aversion (RTPA)/ Real‐Time Place Preference (RTPP)

Real‐Time Place Aversion (RTPA) was performed 3 weeks after surgery, both ChrimsonR‐expressing mice and their controls were bilaterally implanted with optical fibers above the BLA. Mice were placed into the behavioral arena (32 cm × 32 cm) containing two chambers. Mice were habituated to the chamber for 10 min before starting a testing session. We designated one counterbalanced side as the stimulation site for the following 10‐min test. At the beginning of the test session, the mouse was placed on the non‐stimulated side of the chamber, and every time the mouse crossed to the stimulation site, 40 Hz laser stimulation was given until the mouse crossed back into the non‐stimulation side. The movements of mice were tracked by a video camera located above the chamber and analyzed using Smart 3 software. The avoidance score was determined as (Time on Laser ON Side ÷ Total Time) × 100.

#### 2.8.2 Chronic social defeat stress

Chronic social defeat stress was conducted as defined previously.^29^ Briefly, before the experiment, retired male breeder CD1 mice were assessed on three consecutive days to validate their aggressive traits. Subsequently, each experimental male C57BL6/JL mouse (intruder) was placed into the home cage of a novel aggressive CD1 mouse (resident) for 5–10 min for physical defeat for consecutive 10 days. Following the physical defeat, experimental mice were maintained in sensory contact for 24 h by being placed on the opposite side of CD 1 aggressors using a perforated Plexiglass partition. After CSDS, mice were housed individually, and a SIT was conducted 24 h later. Control mice were kept in pairs on opposite sides of the perforated Plexiglass partition in the same cage for 10 days without any physical defeat.

#### 2.8.3 Social interaction

Social avoidance behavior was assessed corresponding to a two‐trail SIT. In the first trial, mice were introduced in an open‐field chamber (44 × 44 × 44 cm^3^) including an empty cage made of mesh wire and plastic sheets (10 × 6 × 8 cm^3^). During behavioral experiments, the mice were tracked with Debut video‐tracking software. Time spent in the social interaction zone (8 cm around the cage) was quantified individually. In the first trial, the time spent in the interaction zone was named “No target.” Mice were then put back in the home cage for 1 min. In the second trial, a novel, CD1 aggressor mouse was put in the cage and was named (Target). The social interaction ratio was evaluated by (time spent in interaction zone with Target/time spent in the interaction zone with No target). Animals were regarded as susceptible when this ratio was <1 and resilient when the ratio was >1.

### 2.9 Two‐trial subthreshold social defeat stress

A two‐trial subthreshold social defeat (SSDS) stress experiment was carried out as defined previously.^30^ The experimental mouse was placed into the home cage of an aggressive CD1 for 10 min for physical defeat. Following 10 min of physical stress, the experimental mice underwent 15 min of sensory stimuli opposite side of CD1 by using a perforated Plexiglass wall. Then the mice underwent a second trial of physical defeat with a novel CD1 mouse and then returned to the home cage, and behavior tests were conducted the following days to evaluate the depressive‐like behavior.

#### 2.9.1 Sucrose preference test

Following the SIT, mice were habituated for two consecutive days to 50 mL tubes with rubber stoppers with ballpoint steel sipper tubes (two‐bottle choice). Subsequently, one bottle was changed with 1% sucrose to determine the sucrose preference. Both bottles including water and sucrose were weighed at several time points, 09:00, 15:00, and 18:00. The bottle's position was changed each time after weight measurements (left to right, right to left) to confirm that the mice do not develop the side preference. Sucrose preference was determined as the percentage (amount of sucrose consumed × 100 (bottle a)/total volume consumed (bottles a + b)). Total sucrose intake over the 24 h was quantified and used to find sucrose preference.

#### 2.9.2 Tail‐suspension test

Briefly, mice were hanged by their tails with adhesive tape (17 cm in length, 1 cm from the tail tip) and approximately 50 cm higher than the surface so no contact could be made. Plastic tubes were positioned on the tails to guarantee mice could neither climb nor hang on to their tail. The mice were with a video camera for 6‐min and the immobile duration of over 6‐min was used to determine the despair‐like behavior.

#### 2.9.3 Open‐field test

Mice were separately presented to the central zone of an open‐field (44 × 44 cm^2^) arena with dim light in the dark phase for 10 min. Their movements were recorded by video camera and analyzed with smart 3.0 software. Anxiety‐like behavior was determined by the time spent in the central zone.

### 2.10 Quantification and statistical analyses

All the data are shown as means ± s.e.m. Statistical evaluations were achieved using GraphPad Prism 8.0.1. software with suitable inferential methods. Normally distributed data were assessed by paired, and unpaired *t*‐tests for two‐group comparisons, one‐ and two‐way analysis of variance (ANOVA), and multiple comparisons were performed by the Bonferroni test. Statistical significance was set at *p < 0.05, **p < 0.01, ***p < 0.001, and ****p < 0.0001 (Table S1).

## 3 RESULTS

### 3.1 Aversive stimuli induced BLA neuronal activities

**FIGURE 1:**
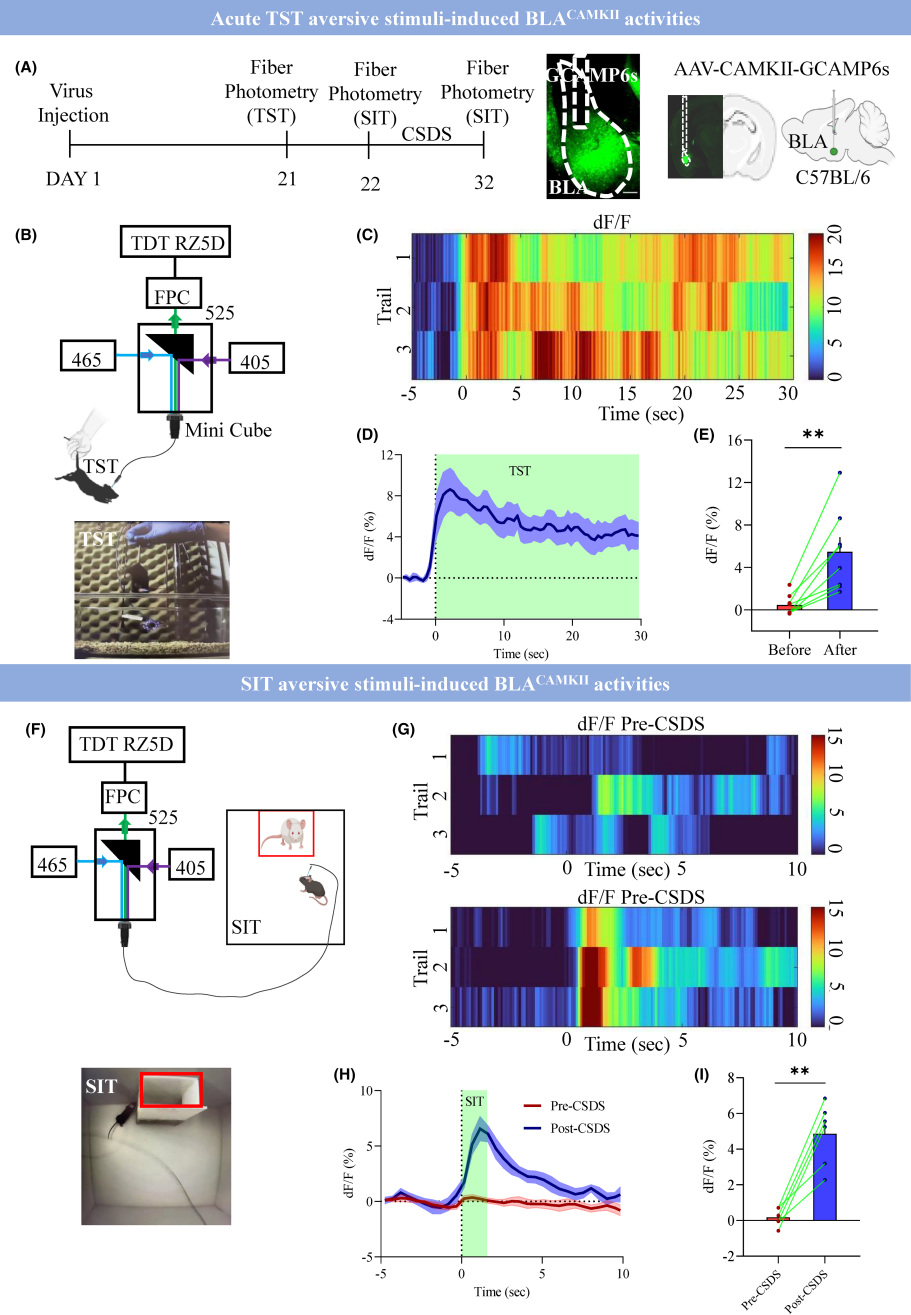
Aversive stimuli‐induced BLA^CAMKII^ neuronal activities. (A) Left: Experimental design, On Day 1, mice were injected with the AAV‐CAMKII‐GCAMP6s virus, and 3 weeks later on Day 21 during the TST, On Day 22, during SIT (Pre‐CSDS) and Day 32 SIT (Post‐CSDS) fiber photometry recordings were performed. Right: representative image showing GCAMP6s expression in the CAMKII neurons of BLA. (B) Schematic representation of experimental setup for fiber photometry for TST. (C) Heatmap showing responses with TST. (D) Average response traces in TST. (E) Average and individual responses during the TST period (0–30 s), **p < 0.0026, *t* = 4.55, df = 7, trails *n* = 24 in *N* = 8 mice, two‐tailed paired *t*‐test. (F) Schematic representation of experimental setup for fiber photometry for SIT. (G) Heatmap showing responses with SIT. (H) Average response traces in SIT. (I) Average and individual responses during the SIT period (0–2 s), **p < 0.0013, *t* = 6.44, df = 5, trail *n* = 18 in *N* = 6 mice. All data are shown as mean ± s.e.m.

To investigate how the aversive stimuli modulate the BLA neuronal activities, we adopted a well‐established TST^27^, ^31^ and chronic social defeat stress (CSDS) paradigm‐induced social avoidance task.^32^ First, we found that the CSDS decreased the social interaction time in the presence of the target (Figure S1A,B; control 85.24 ± 3.07 vs. CSDS 45.6 ± 9.08; *p* < 0.0004) and increased the c‐fos activity in the BLA compared with control mice (Figure S1C,D; control 5.52 ± 0.90 vs. CSDS 21.71 ± 2.22; *p* < 0.0001). Next, we performed fiber photometry^33^ to directly track the BLA^CAMKII^ neuronal activity during the TST and SIT. Our fiber photometry setup employed a 465 nm beam to trigger Ca^2+^‐dependent GCaMP6 fluorescence, as well as a 405‐nm beam to view an isosbestic reference signal, which balances photo‐bleaching and movement‐related artifacts. The genetically encoded calcium indicator GCaMP6s was expressed in the BLA^CAMKII^ neurons via injecting AAV9‐CAMKII‐GCaMP6s into the BLA of C57 mice. The fiber photometry recordings were performed on day 21 during the TST, on day 22 before CSDS (pre‐CSDS). Then mice underwent 10 days of CSDS and on Day 32 again fiber photometry recording was performed during SIT Post‐CSDS (Figure 1A). We observed the tail suspension aversive stimuli robustly activated BLA^CAMKII^ neurons as indicated by increased calcium responses from 0.57 ± 0.37 to 5.34 ± 1.3 during TST (Figure 1B–E; *p* < 0.003). Footshock (FS) is another aversive stimuli that also evoked strong calcium responses from −0.37 ± 0.42 to 10.45 ± 1.71 (Figure S1E,F; *p* < 0.0003). Moreover, we found that the BLA^CAMKII^ neurons showed increased activity during the aversive social stimuli as exhibited by elevated calcium responses from 0.22 ± 0.21 to 5.1 ± 0.73 during the SIT (Figure 1F–I; *p* < 0.0013).

### 3.2 Effect of laser activation of BLA^CAMKII^ or BLA^GABA^ on real‐time behavior

**FIGURE 2:**
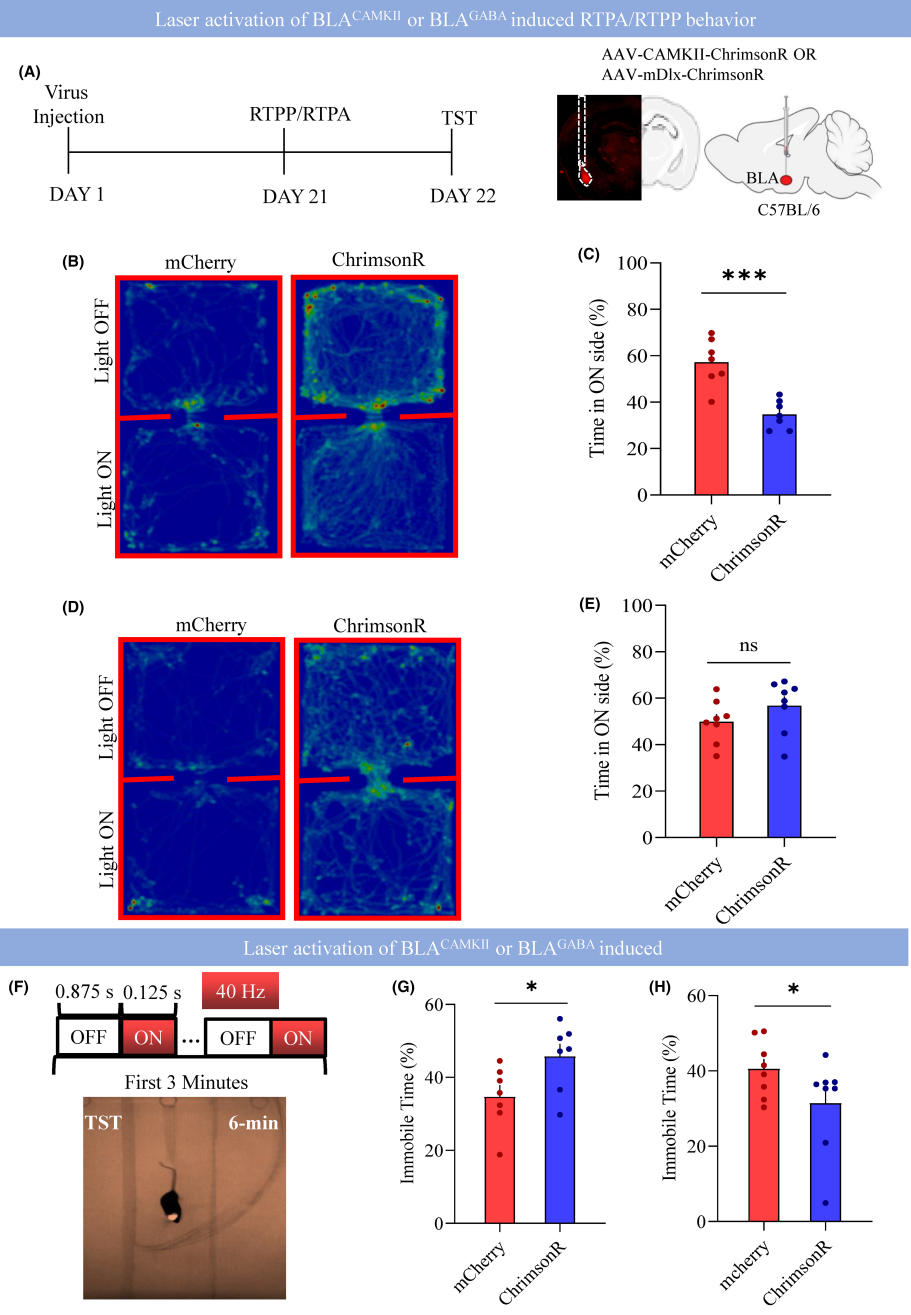
Effect of laser activation of BLA^CAMKII^ or BLA^GABA^ on real‐time behavior. (A) Left: Experimental design, Day 1 virus injection, Day 21 RTPP/RTPA test, and Day 22 TST. Right: representative image showing the location of fiber tips and virus expression in the BLA. (B) Heat map showing the time spent in light on the paired chamber and light off chamber between mcherry and CAMKII‐chrimsonR group. (C) % Time spent on light ON side between mcherry and CAMKII‐chrimsonR group, ***p < 0.0003, *t* = 4.985, df = 12, *N* = 7 mice, two‐tailed unpaired *t*‐test. (D) Heat map showing the time spent in light on the paired chamber and light off chamber between mcherry and Dlx‐chrimsonR group. (E) % Time spent on light ON side between mcherry and Dlx‐chrimsonR group, *p* < 0.2, *t* = 1.335, df = 14, *N* = 8 mice, two‐tailed unpaired *t*‐test. (F) Experimental setup for laser stimulation and TST. (G) % Immobile time during tail suspension test mcherry and CAMKII‐chrimsonR, *p < 0.0381, *t* = 2.330, df = 12, *N* = 7 mice, two‐tailed unpaired *t*‐test. (H) % Immobile time during tail suspension test mcherry and Dlx‐chrimsonR, *p < 0.0497, *t* = 1.765, df = 14, *N* = 8 mice, one‐tailed unpaired *t*‐test. All data are shown as mean ± s.e.m.

We have shown above that the aversive stimuli led to the hyperactivity of BLA via increased BLA^CAMKII^ neuronal activity. The next experiment examined the effect of laser stimulation of BLA^CAMKII^ or BLA^GABA^ on real‐time behavior.^6^, ^34^ We injected ChrimsonR with a specific promoter, CAMKII for glutamatergic neurons or Dlx for GABAergic neurons into BLA and implanted optic fiber over the BLA to specifically activate either BLA^CAMKII^ or BLA^GABA^ neurons soma in awake, freely moving mice (Figure 2A). Laser stimulation of BLA^CAMKII^ neurons reduced the time spent in the light‐paired chamber in real‐time place aversion (RTPA) task (Figure 2B,C; mCherry 57.22 ± 3.87 vs. ChrimsonR 34.70 ± 2.33; *p* < 0.0003) and increased the immobile time during the TST (Figure 2F,G; mCherry 34.62 ± 3.24 vs. ChrimsonR 45.72 ± 3.50; *p* < 0.04). However, stimulation of BLA^GABA^ neurons decreased the immobile time during the TST (Figure 2F–H; mCherry 40.51 ± 2.67 vs. ChrimsonR 31.39 ± 4.42; *p* < 0.05) but induced no significant difference in time spent in the light‐paired chamber in real‐time place preference (RTPP) task compared with the control group (Figure 2D,E; mCherry 49.91 ± 3.26 vs. ChrimsonR 56.81 ± 4.01; *p* < 0.02).

### 3.3 BLA^CAMKII^ neurons mediate depressive‐like behaviors

**FIGURE 3:**
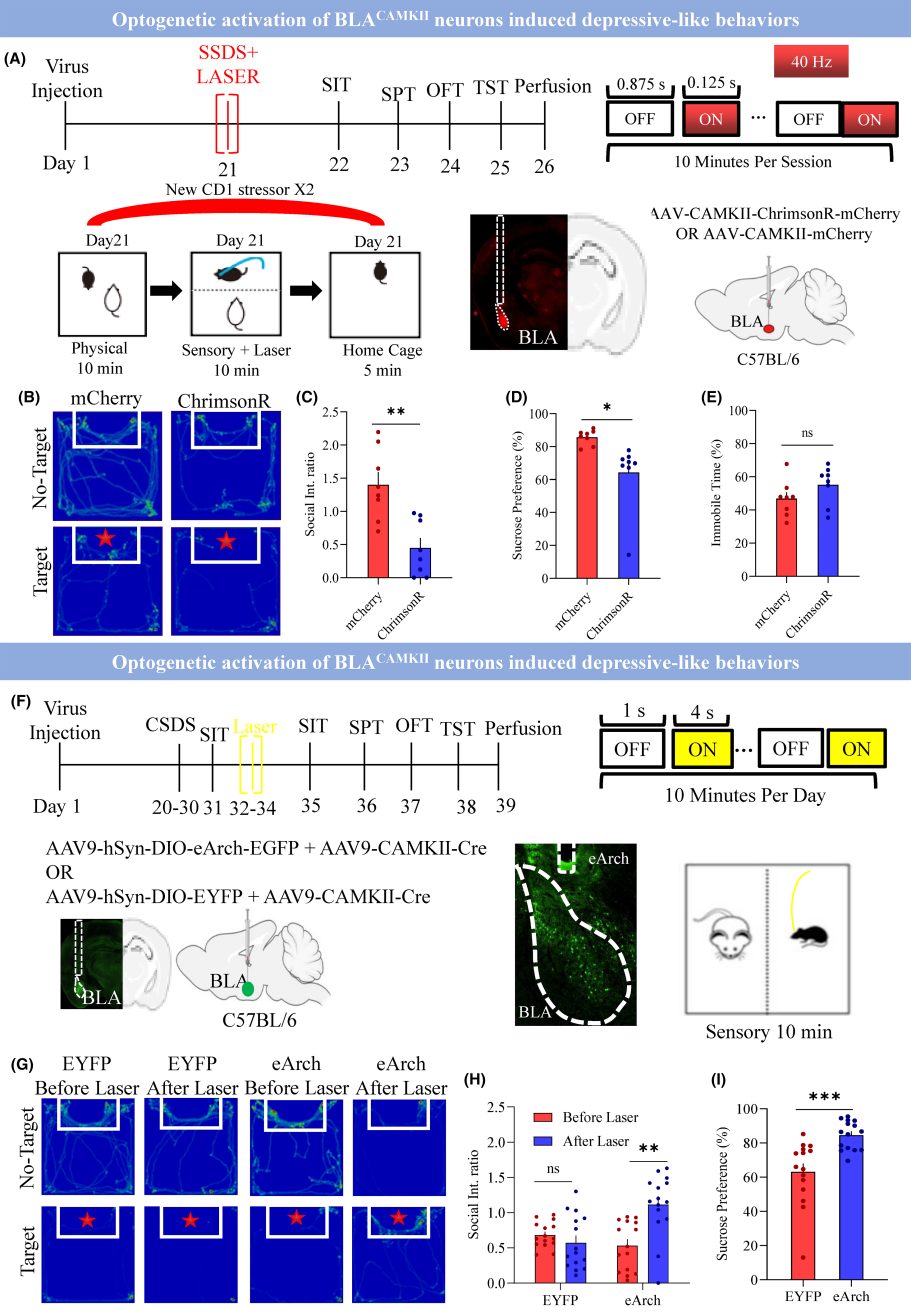
BLA^CAMKII^ neuron suppression relieves depressive‐like behavior. (A) Schematic illustration of experimental design and virus expression. Day 1 virus injection, Day 21 SSDS with laser stimulation, Day 22–25 behaviors, and Day 26 perfusion. (B) Showing the heat map of mcherry versus ChrimsonR in the presence of No‐target and Target. (C) Social interaction ratio mCherry versus ChrimsonR, **p < 0.0014, *t* = 3.97, df = 14, *N* = 8, two‐tailed unpaired *t*‐test. (D) Sucrose preference mCherry versus ChrimsonR, *p < 0.012, *t* = 2.89, df = 14, *N* = 8, two‐tailed unpaired *t*‐test. (E) % Immobile time during TST mCherry versus ChrimsonR, *p* < 0.16, *t* = 1.49, df = 14, *N* = 8, two‐tailed unpaired *t*‐test. (F) Top: Schematical illustration of the experimental design. Day 1 virus injection, Day 20–30 CSDS, Day 31 SIT, Day 32–34 sensory stress with laser stimulation, Day 35–38 behaviors, and Day 39 perfusion. Bottom: Showing the virus expression and a fiber track. (G) Showing the heat map of EYFP versus eArch in the presence of Non‐target and Target. (H) Average social interaction ratio of EYFP versus eArch group, RM two‐way ANOVA, Interaction: *p* < 0.0031, F(1, 28) = 10.49, Row factor: *p* < 0.0116, F(1, 28) = 7.289, Column factor: *p* < 0.0339, F(1, 28) = 4.977, EYFP before laser versus EYFP after laser: *p* < 0.9642, *t* = 0.7124, df = 28, eArch before laser versus eArch after laser: **p < 0.0012, *t* = 3.87, df = 28. Adjustment: Bonferroni *N* = 15 mice. (I) % Sucrose preference between EYFP versus eArch group, ***p < 0.0004, *t* = 4.052, df = 28, *N* = 15 mice, two‐tailed unpaired *t*‐test. All data are shown as mean ± s.e.m.

It is known that CSDS induced depressive‐like behaviors,^35^, ^36^ but SSDS could not induce depressive‐like behavior unless it is combined with chemogenetic/optogenetic manipulation of stress‐mediating neurons.^30^, ^36^ Consistent with previous studies,^30^ we found that the SSDS could not induce depressive‐like behavior but CSDS induced the depressive‐like behavior which is indicated by decreased social interaction ratio, decreased sucrose preference, and center time in the open field along with increased immobile time in the TST (Figure S2A–E). The following experiment examined whether optogenetic stimulation of BLA^CAMKII^ neurons with subthreshold social defeat stress (SSDS) could induce depressive‐like behavior (decreased social interaction, decreased sucrose preference, increased immobile time), in the mice (Figure 3A). We applied the laser stimulation during a 10‐min sensory period following a physical defeat. We successfully induced decreased social interaction (Figure 3B,C; mCherry 1.40 ± 0.19 vs. ChrimsonR 0.45 ± 0.15; *p* < 0.0014), decreased sucrose preference (Figure 3D; mCherry 85.73 ± 1.61 vs. ChrimsonR 64.29 ± 7.24; *p* < 0.012), and trend to increased immobile time in TST (Figure 3E; mCherry 46.78 ± 3.84 vs. ChrimsonR 55.10 ± 4.08; *p* < 0.1). However, there was no significant change in center time in the open field test (OFT) (Figure S3A–C; mCherry 11.59 ± 1.06 vs. ChrimsonR 11.15 ± 1.67; *p* < 0.83). The c‐fos activity was also increased in the ChrimsonR group compared with the control mCherry group (Figure S3D,E; ChrimsonR vs. mCherry, 28.14 ± 2.9 vs. 12.75 ± 1.4, *p* < 0.0001).

Chronic aversive stimuli experiencing is a fundamental risk factor that leads to the development of depressive‐like states. Therefore, we next adopted a CSDS model to further explore whether BLA^CAMKII^ neurons mediate stress‐induced depressive‐like behavior, we silenced these neurons by expressing inhibitory opsin eArch (Figure 3F). Preclinical and clinical studies showed that chronic treatment of antidepressants exhibits relief from depression.^37^, ^38^, ^39^, ^40^ Chronic therapy could be the best way to treat depression. Thus, we adopted the three consecutive days of optogenetic inhibition of BLA^CAMKII^ manipulation to treat the depressive behaviors in the mice to achieve the best outcome. We found that optogenetic inhibition of eArch expressing neurons reduced stress susceptibility in the SIT (Figure 3G,H; EYFP before laser 0.68 ± 0.05 vs. EYFP after laser 0.58 ± 0.10; *p* < 0.96; eArch before laser 0.53 ± 0.09 vs. eArch after laser 1.12 ± 0.12; *p* < 0.0012), increased sucrose intake in SPT (Figure 3I; EYFP 63.11 ± 4.82 vs. eArch 84.55 ± 2.18; *p* < 0.0004), reduced immobility in the TST (Figure S3F; EYFP 47.78 ± 3.94 vs. eArch 24.63 ± 2.54; *p* < 0.0001), however, there was no improvement in center time in OFT (Figure S3G–I; EYFP 6.21 ± 0.77 vs. eArch 5.78 ± 1.03; *p* < 0.74). Optogenetic inhibition of BLA^CAMKII^ neurons not only inhibited the depressive‐like states but also reduced the c‐fos activity in the BLA (Figure S3J,K; EYFP 24.91 ± 2.20 vs. eArch 13.88 ± 1.30; *p* < 0.0004). Together these results of activation and inactivation experiments suggest that the BLA^CAMKII^ neurons mediate the expression of physical stress‐induced depressive‐like behavior.

### 3.4 BLA^GABA^ modulates the BLA^CAMKII^ neuronal activities and depressive‐like behaviors

**FIGURE 4:**
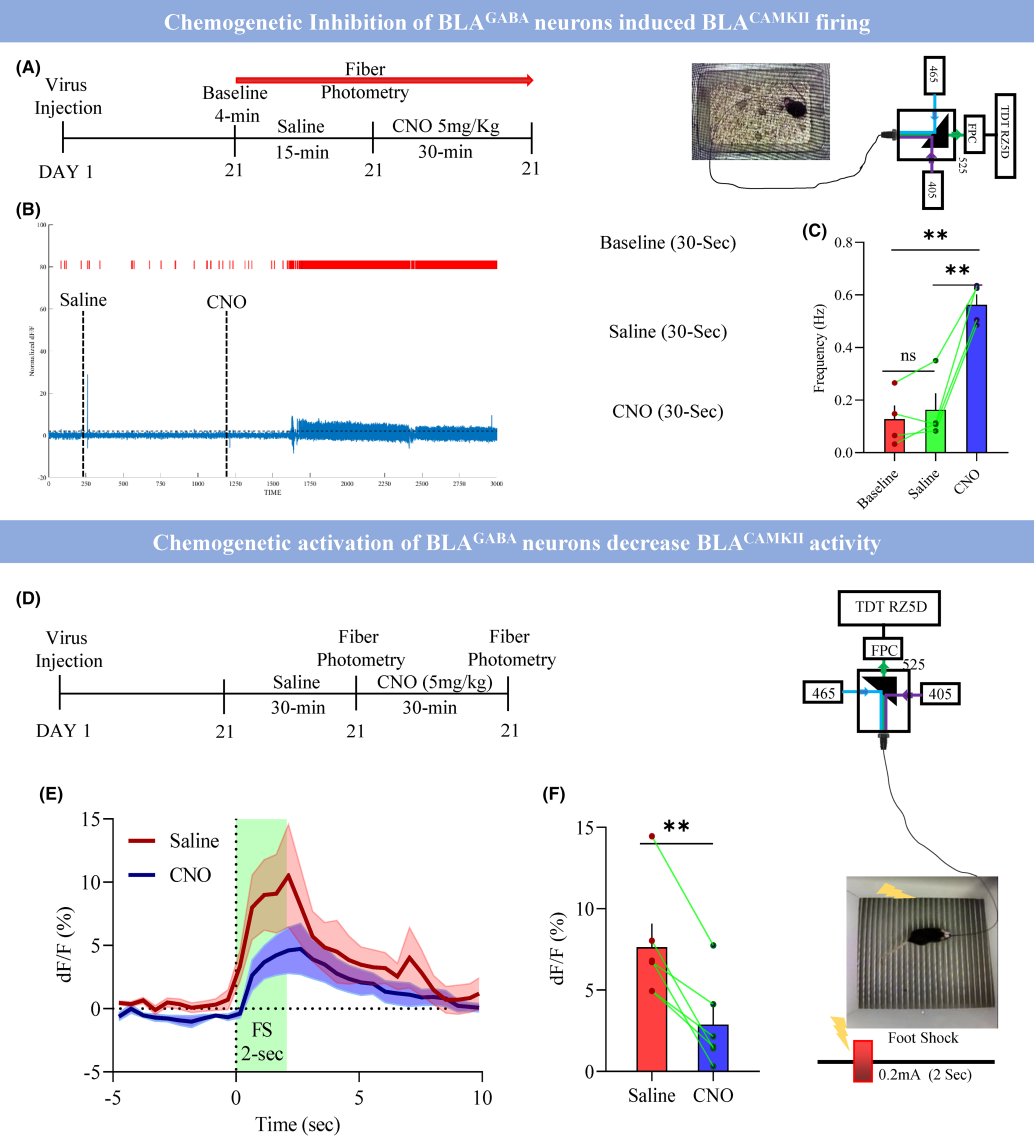
BLA^GABA^ modulates the BLA^CAMKII^ neuronal activities. (A) Schematical illustration of the experimental design and setup. Day 1 virus injection, Day 21 fiber photometry recording, 4‐min baseline, 15‐min after saline, then 30‐min after CNO. (B) Normalized change in calcium response and spike at baseline, after saline and CNO. (C) Firing frequency across baseline, saline, and CNO, RM one‐way ANOVA, Treatment between columns: *p* < 0.0013, F(1.414, 4.241) = 51.85, Individual between rows, *p* < 0.04, F(3, 6) = 5.24, Baseline versus Saline, *p* < 0.53, *q* = 1.71, df = 3, Baseline versus CNO, **p < 0.0099, *q* = 10.67, df = 3, Saline versus CNO, **p < 0.0086, *q* = 11.18, df = 3, Adjustment: Tukey's, *n* = 12 trails in *N* = 4 mice. (D) Illustration of experiment design and setup. Day 1 virus injection, Day 21 fiber photometry recording, 30 min after saline, then 30 min after CNO. (E) Average response traces of BLA^CAMKII^ with saline versus CNO. (F) Change in calcium responses (%) (0–2 s), saline versus CNO, **p < 0.0022, *t* = 5.74, df = 5, trails *n* = 18 in *N* = 6 mice, two‐tailed paired *t*‐test. All data are shown as mean ± s.e.m.

Then, we explored how the BLA^GABA^ neurons control the BLA^CAMKII^ neurons' activities. We injected mDlx‐hM4Di and CAMKII‐GCAMP6s viruses into BLA and performed fiber photometry recording after recovery (Figure 4A). CNO injection increased the firing of BLA^CAMKII^ neurons as indicated by the increased number of spikes (Figure 4B) and increased firing frequency of BLA^CAMKII^ neurons (Figure 4C; Baseline 0.13 ± 0.05 vs. Saline 0.16 ± 0.06 vs. CNO 0.56 ± 0.04; *p* < 0.009) as compared with baseline and saline recording duration. In a separate group of mice, we injected mDlx‐hM3Dq and CAMKII‐GCAMP6s viruses into BLA to examine whether the chemogenetic activation of BLA^GABA^ could inhibit the BLA^CAMKII^ neuronal activity. The saline was injected 30 min before fiber photometry recording and looked at the foot shock‐induced (0.2 mA) BLA^CAMKII^ neuronal activity then CNO was injected and 30 min later again fiber photometry recording was performed (Figure 4D). It has been found that CNO injection decreased the foot shock‐induced BLA^CAMKII^ neuronal activity as shown by decreased calcium responses after CNO application (Figure 4E,F; Saline 8.46 ± 2.25 vs. CNO 2.88 ± 1.09; *p* < 0.002).

**FIGURE 5:**
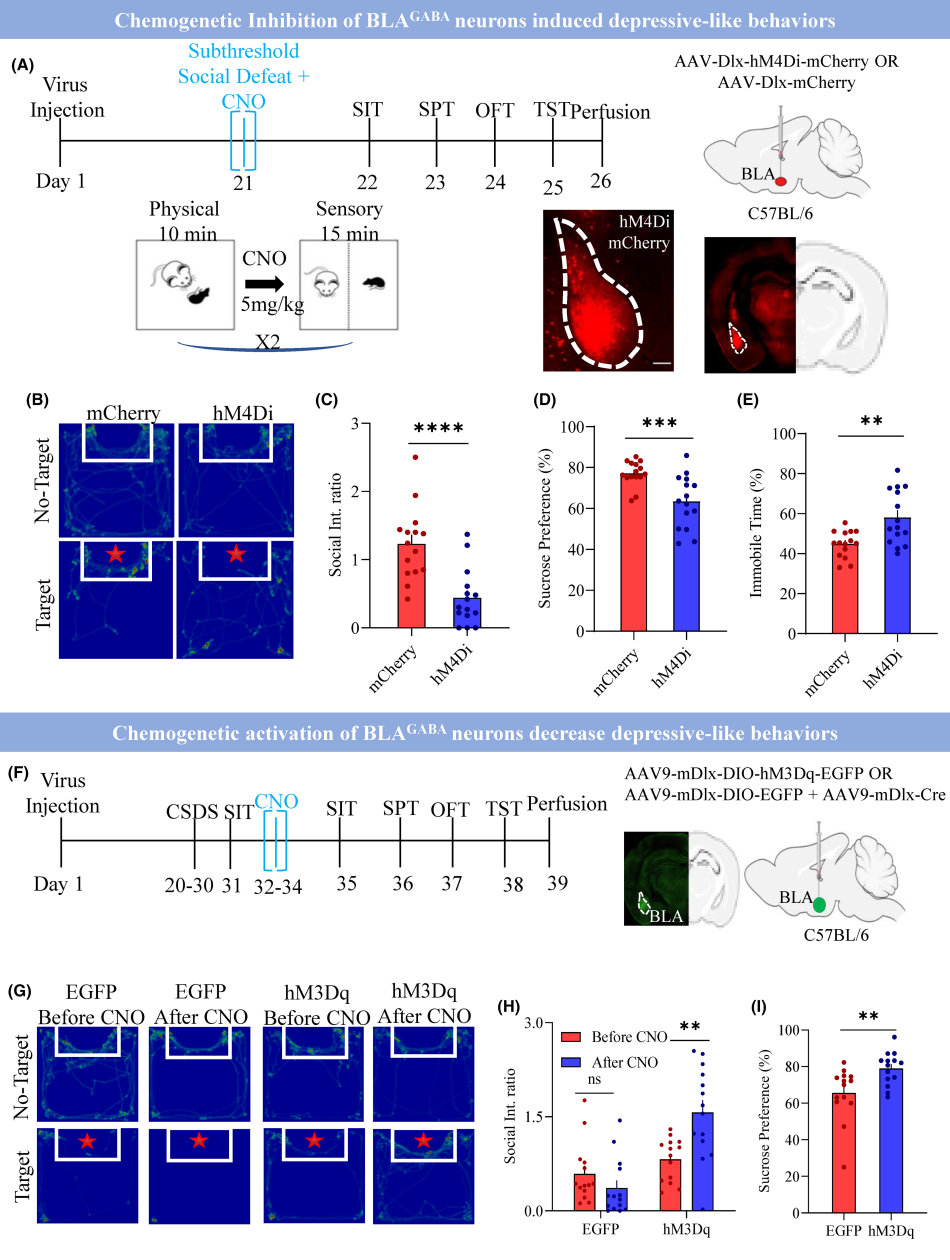
BLA^GABA^ modulates depressive‐like behaviors. (A) Top: Schematical illustration of the experimental design. Day 1 virus injection, Day 21 SSDS with laser stimulation, Day 22–25 behaviors, and Day 26 perfusion. Right bottom: showing the virus expression in hM4Di‐mCherry and injection site. (B) Showing the heat map of hM4Di versus mCherry control in the presence of Non‐target and Target. (C) Average social interaction ratio of hM4Di versus mCherry control group, ****p < 0.0001, *t* = 4.53, df = 28, *N* = 15 mice, two‐tailed unpaired *t*‐test. (D) % Sucrose preference between hM4Di versus mCherry control group, ***p < 0.0008, *t* = 3.76, df = 28, *N* = 15 mice, two‐tailed unpaired *t*‐test. (E) Immobile duration during the tail suspension test between hM4Di versus mCherry control group, **p < 0.0021, *t* = 3.39, df = 28, *N* = 15 mice, two‐tailed unpaired *t*‐test. (F) Left: Schematically representation of an experimental design. Day 1 virus injection, Day 20–30 CSDS, Day 31 SIT, Day 32–34 sensory stress with laser stimulation, Day 35–38 behaviors, and Day 39 perfusion. Right: Showing the virus expression and injection site. (G) Showing the heat map of EGFP and hM3Dq groups in the presence of Non‐target and Target before CNO administration and after CNO administration. (H) Average social interaction ratio of EGFP and hM3Dq groups, RM Two‐way ANOVA, interaction: *p* < 0.0036, F(1, 26) = 10.25, Row factor: *p* < 0.0001, F(1, 26) = 33.46, Column factor: *p* < 0.098, F(1, 26) = 2.94, EGFP before CNO versus EGFP after CNO: *p* < 0.61, *t* = 1.05, df = 26, hM3Dq before CNO versus hM3Dq after CNO: *p < 0.0036, *t* = 3.48, df = 26, Adjustment: Bonferroni *N* = 14 mice. (I) % Sucrose preference between EGFP and hM3Dq groups, **p < 0.0073, *t* = 2.91, df = 26, *N* = 14 mice, two‐tailed unpaired *t*‐test. All data are shown as mean ± s.e.m.

Afterward, we investigated the role of BLA^GABA^ neurons in depression at the behavior level using chemogenetic inhibition. We injected the Dlx‐hM4Di or Dlx‐mCherry virus into the BLA of C57 mice. Mice underwent a two‐trail sub‐threshold social defeat paradigm. Immediately after the first trial of physical defeat, mice received a single injection of 5 mg/kg CNO and were kept in a cage opposite to (facing) the CD‐1 mouse for 15‐min sensory stimulation, followed by a second trial of physical defeat (Figure 5A). Chemogenetic inhibition of BLA^GABA^ neurons facilitated the induction of depressive‐like behavior with SSDS in mice. We found that chemogenetic inhibition of BLA^GABA^ neurons induced stress susceptibility in the SIT (Figure 5B,C; mCherry 1.23 ± 0.14 vs. hM4Di 0.44 ± 0.11; *p* < 0.0001), decreased performance in the SPT (Figure 5D; mCherry 77.14 ± 1.57 vs. hM4Di 63.33 ± 3.32; *p* < 0.0008), increased immobility in the TST (Figure 5E; mCherry 44.82 ± 1.72 vs. hM4Di 58.02 ± 3.50; *p* < 0.002), and decreased center time in OFT (Figure S4A–C; mCherry 15.82 ± 1.29 vs. hM4Di 7.02 ± 0.91; 0.0001). Chemogenetic inhibition of BLA^GABA^ neurons during SSDS not only facilitated the induction of depressive‐like states but also increased the c‐fos activity in the BLA (Figure S4D,E; mCherry 8.18 ± 1.12 vs. hM4Di 20.67 ± 2.21; *p* < 0.0001).

If hyperactivity of the BLA is characteristic of susceptibility to stress, we hypothesized that chemogenetic activation of BLA^GABA^ could promote resilience to social stress. To test this, we selectively expressed either hM3Dq‐EGFP or only EGFP in BLA^GABA^ neurons (Figure 5F). Chronically chemogenetic activation of the BLA^GABA^ neurons over three consecutive days in the defeated susceptible mice expressing hM3Dq‐EGFP showed an increase in social interaction (Figure 5G,H; Before CNO hM3Dq 0.82 ± 0.09 vs. After CNO hM3Dq 1.57 ± 0.20; *p* < 0.003), as well as a dramatic improvement in the SPT (Figure 5I; EGFP 65.51 ± 3.94 vs. hM3Dq 78.98 ± 2.43; *p* < 0.007), immobility time in the TST (Figure S4F; EGFP 51.60 ± 5.29 vs. hM3Dq 33.77 ± 2.45; *p* < 0.0051), and increased center time in the OFT (Figure S4G–I; EGFP 9.29 ± 1.15 vs. hM3Dq 14.26 ± 1.35; *p* < 0.009). However, the same 3‐day chemo manipulation in the EGFP group had no effect. These results suggest an antidepressant effect caused by 3‐day chronic chemo manipulation of the BLA^GABA^ neurons in susceptible mice. Additionally, we observed that the chemogenetic stimulation of BLA^GABA^ reduced the c‐fos activity in the BLA (Figure S4J,K; EGFP 17.29 ± 2.26 vs. hM3Dq 8.81 ± 1.20; *p* < 0.003). In summary, BLA^GABA^ modulates depressive‐like behavior.

### 3.5 Input sources to the basolateral amygdala

What are the input structures of BLA neurons? To determine the input sources to the BLA, we injected the retrograde Retro‐AAV‐Cre virus into the BLA of Ai14 mice. Retrogradely infected cells were detected in several regions, in the particular inferior limbic cortex (IL), prelimbic cortex (PL), anterior cingulate cortex (ACC), auditory cortex (Auc), anterior insular cortex (AIC), and lateral entorhinal cortex (LEnt) (Figure S5A–C). In general, these input sources are consistent with previous anatomical data,^41^ These structures have been implicated in stress responses, and anxiety,^18^, ^42^, ^43^, ^44^, ^45^ supporting that BLA may integrate aversive events information from multiple sources.

## 4 DISCUSSION

Although there is rising interest in the role of BLA in depression states, how aversive stimuli dynamically alter BLA function remains elusive. To cover all facets, we combined fiber photometry, immediate early gene expression (c‐fos), chemo‐genetics, optogenetics, behavioral analysis, and anatomy to present multiple lines of evidence to show the crucial role of BLA neurons in depression. By assessing the scale of neuronal activation of BLA^CAMKII^ with aversive stimuli, we presented comprehensive evidence that exposure to aversive stimuli activates the BLA^CAMKII^ neurons. Cell‐type‐specific investigation disclosed opposite roles of BLA^CAMKII^ and GABAergic neurons in regulating depressive‐like behavior: CAMKII neurons enhance depressive‐like behavior and mediate stress‐induced depressive states, whereas the GABAergic neurons suppress depressive‐like behaviors. The GABAergic neurons antagonize the effects of CAMKII neurons at the neural as well as behavioral levels. Besides, we demonstrated that BLA receives input from several structures which are previously implicated in stress responses, and anxiety. These results, for the first time to our knowledge, implicate how BLA individual neurons modulate depressive states.

The amygdala is a crucial brain region for the management of stress and stress hormones on emotional behaviors.^46^, ^47^, ^48^, ^49^ In particular, stressors can impact amygdala‐dependent fear and depressive‐like behavior.^50^, ^51^, ^52^, ^53^ Hyperactivity of the basolateral amygdala during stressful event re‐experience and in depression is one of the most reliable findings of clinical^20^, ^21^ and pre‐clinical neuroscience.^54^, ^55^ Although BLA has been implicated in the regulation of emotional behavior, the functional role of BLA^CAMKII^ neurons is still unclear. Consistent with previous studies, we found that aversive stimuli increased the activity of BLA. Of note, the tail suspension aversive stimuli and chronic social defeat‐induced SIT aversive stimuli dramatically magnified the activity of BLA^CAMKII^ neurons. Activation of these neurons triggers real‐time place aversion and despair‐like behavior, while suppressing these neurons alleviates the expression of depressive‐like behavior after exposure to chronic social stress. The increased activity of CAMKII neurons after exposure to stress accounts for the persistence of the induced depressive state and suppressing these neuron activities is sufficient to reduce the basal‐level depressive state. Together, our results manifest that BLA^CAMKII^ neurons regulate the induction, expression, and maintenance of depressive states in general.

The regulation of negative emotional states including fear, anxiety, and depression, depends on the processing of negative stimuli. The activation of BLA^CAMKII^ neurons appears to result in an extremely strong negative emotion as compared with the previously studied structures such as the bed nucleus of the stria terminalis (BNST)^56^ and paraventricular nucleus (PVN).^57^ Notably, the percentage of time spent in the photo‐stimulation chamber is comparatively less than when other structures are activated.^56^, ^57^ These results offer strong proof that the activity of BLA^CAMKII^ neurons can encode extremely negative valence, and may thus lead to relentless fear and a depressive state. This property of BLA^CAMKII^ neurons manifests that they might be a unique therapeutic target for treating severe depressive disorders.

Opposite to the CAMKII neurons, our experiments demonstrate that the BLA^GABA^ neurons encode positive valence and promote antidepressant‐like effects. Interestingly, by comparing CAMKII and GABAergic neurons, our current experiments reveal that the antidepressant‐like effect caused by the activation of the GABAergic neurons is much stronger than suppressing the CAMKII neurons. In addition to antidepressant‐like effects, activation of GABAergic neurons is also anxiolytic which is consistent with previous findings showing the impaired decreased GABAergic neuron activity associated with anxiety state.^58^ These results suggest that the activity of CAMKII neurons cannot encode a full scale of valence values and positive effects are primarily coded by GABAergic neurons.

Our results indicated that the inhibition of BLA^GABA^ increased the firing frequency of BLA^CAMKII^ neurons. Besides, we found that the foot shock increased the activity of BLA^CAMKII^, whereas chemogenetic stimulation of BLA^GABA^ neurons reduced the activity of BLA^CAMKII^ neurons. These results supported that BLA^CAMKII^ neuron activity is tightly controlled by local GABAergic interneurons.^19^, ^59^, ^60^ Chemogenetic stimulation of BLA^GABA^ neurons may enhance the inhibition by high‐frequency long‐term activation of BLA GABAergic neurons. This is supported by a study published in the SSRN elibrary (preprint) showing that chemogenetic inhibition decreases the number of seizures in the KA‐induced epilepsy mice model by enhancing GABAergic inhibition.^61^ High‐frequency stimulation of GABA neurons may facilitate the release of inhibitory CCK which strengthen the inhibitory synapses and enhanced the inhibition in the amygdala. Indeed, a recent study showed that the somatodendritic release of CCK enhanced the GABAergic transmission onto VTA dopamine neurons.^62^ Recently, we showed that the high‐frequency stimulation of GABAergic neurons induced iLTP in the auditory cortex, and this effect was mediated by a novel CCK receptor.^22^ Furthermore, our previous studies also showed that high‐frequency stimulation induces the release of CCK,^45^, ^61^, ^63^ which is also consistent with the old theory that proposed that the release of neuropeptides required high‐frequency stimulation.^64^ However, further studies are required to find direct evidence that in the amygdala high‐frequency stimulation causes the release of CCK from inhibitory neurons.

Heightened activity of BLA is associated with anxiety and depression.^20^, ^55^, ^65^ However, whether enhancing the inhibition in the BLA could alleviate depressive‐like behaviors remains unknown. Here, we demonstrated that enhancing the inhibition via high‐frequency stimulation of BLA^GABA^ or directly suppressing BLA^CAMKII^ neurons attenuates the depressive‐like behavior in susceptible mice, while inhibition of BLA^GABA^ neurons or activation of BLA^CAMKII^ tiger the depression.

What causes the hyperactivity of BLA neurons during aversive stimuli and depression? BLA receives input from several sources including mPFC, ACC, PVN, LEnt, and IC (Figure S5), neurons of these structures are known to respond to aversive stimuli,^18^, ^42^, ^43^, ^44^, ^45^ yet it is unclear how these structures modulate the BLA individual neuronal activity, especially during the progression of depression. The BLA neurons could be able to incorporate numerous types of information to persuasively regulate depressive phenotype. Several mechanisms could underlie the enhanced BLA activity after stress exposure: increases in the synaptic strength of the inputs and or spiking activity of input structures.

In sum, our findings demonstrate that BLA^GABA^ and BLA^CAMKII^ neuronal activity are correlated with depressive‐like behavior. The balance among activities of these neurons through competitive interaction may consequently allow the expression of an antidepressant response.

## AUTHOR CONTRIBUTIONS

J.H. and M.A. designed the experiments; M.A. and X.C. collected and analyzed the data for fiber photometry; M.A. and H.W. collected and analyzed the data for the behavioral part and performed immunohistochemistry. M.A. wrote the manuscript. J.H. and X.C. assisted in editing and writing the manuscript. J.H. supervised the project.

## CONFLICT OF INTEREST STATEMENT

All other authors declare they have no financial or competing interests.

## Supporting information


Data S1:

## Data Availability

The data that support the findings of this study are available from the corresponding author upon reasonable request.
