# Supplementary material for: Potentiated GABAergic neuronal activities in the basolateral amygdala alleviate stress‐induced depressive behaviors
Source: CNS Neurosci Ther. 2023 Sep 16;30(3):e14422. doi: 10.1111/cns.14422 (PMC10915993; doi:10.1111/cns.14422)
Supplement: Supplementary file 1 — Data S1: [file CNS-30-e14422-s001.docx]

**Supplementary Material**

**
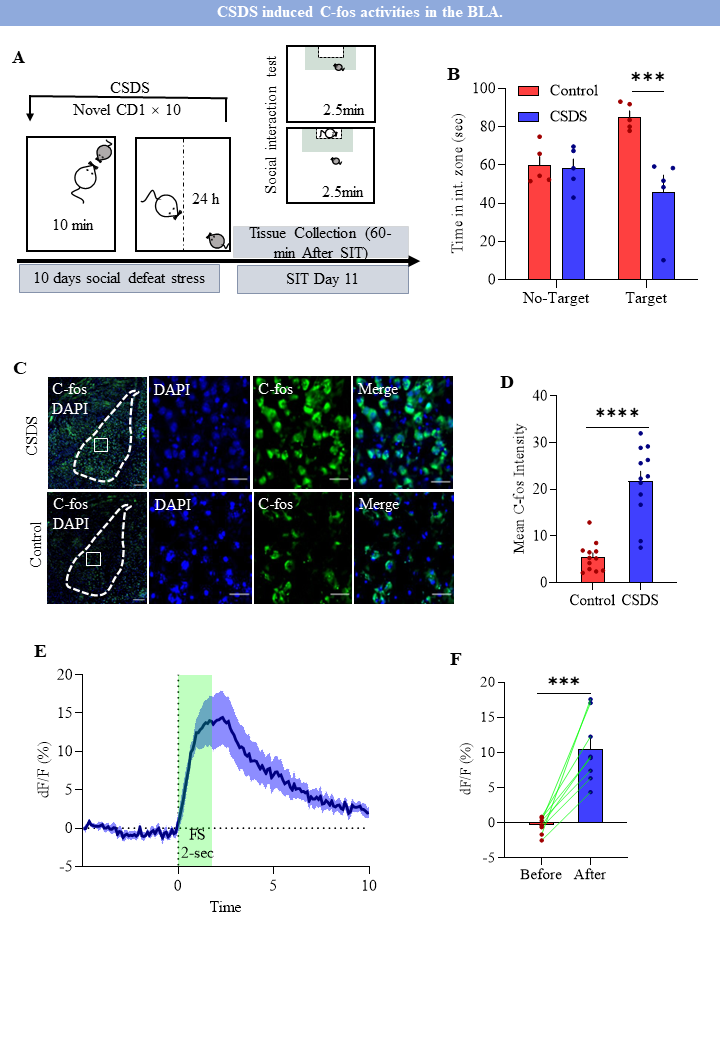
**

**Figure S1: Aversive stimuli activate BLA.**

**A.** Schematic representation of an experimental design. **B.** Time spent in the interaction zone with or without a target, Two-way ANOVA, Interaction: P < 0.0049, F (1, 16) = 10.62, Row factor: P < 0.29, F (1, 16) = 1.2, Column factor: P < 0.002 F (1, 16) = 12.43, Control vs CSDS No-target: P < 0.999, t = 0.19, df = 16, Control vs CSDS target: P < 0.0004, t= 4.87, df = 16, Adjustment: Bonferroni N = 5 mice. **C.** Representative images showing the c-fos activity in the BLA of control and CSDS mice. The scale bar was set to 200 um on the left images and zoomed on the right 50 um. **D.** Showing the mean c-fos intensity in the BLA of control and CSDS mice, P < 0.0001, t = 6.7624, df = 22, Slices n = 12 from N = 3 mice, two-tailed unpaired t-test. **E.** Average response traces of CAMKII with footshock (0.2mA for 2 s). **F.** Average and individual responses before and during the footshock period (0-2 s), P < 0.0003, t = 6.461, df = 7, trails n=24 in N = 8 mice, two-tailed paired t-test. All data are shown as mean ± s.e.m.

**
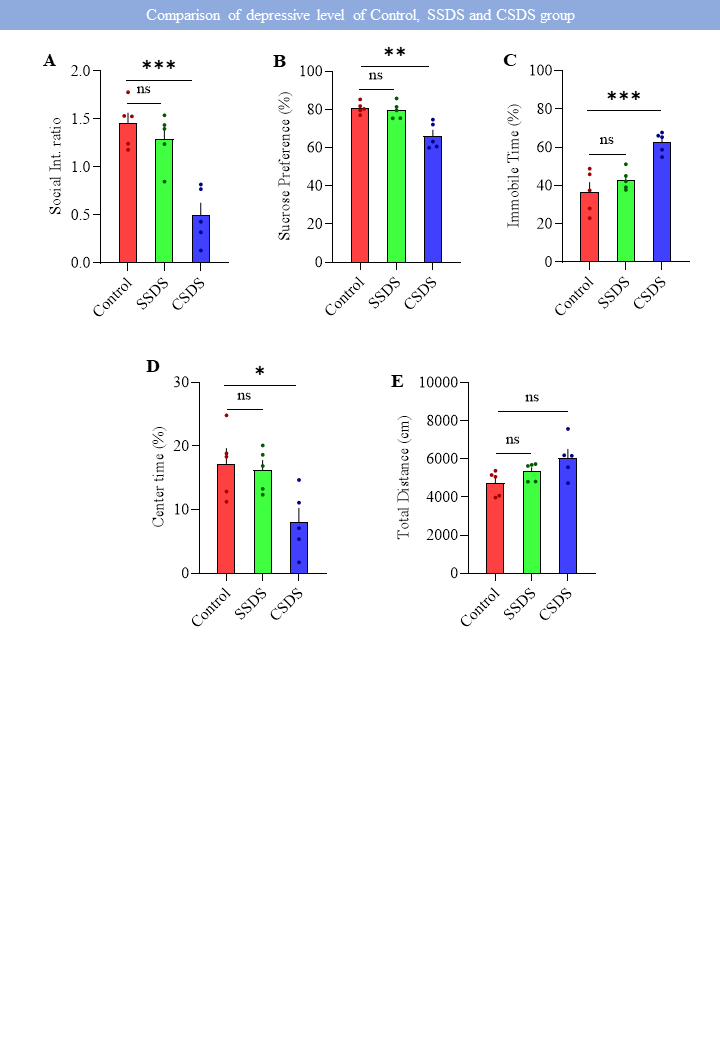
**

**Figure S2: Comparison of depressive levels between control, SSDS, and CSDS groups.**

**A.** Social interaction ratio Control, SSDS, and CSDS: Two-way ANOVA, P < 0.0002, F(2, 12) = 18.05; Control vs SSDS, P<0.99, t = 0.94, df = 12; Control vs CSDS, P<0.0003, t = 5.61, df = 12; Bonferroni’s adjustments. **B.** Sucrose preference Control, SSDS, and CSDS: Two-way ANOVA, P < 0.0009, F(2, 12) = 13.16; Control vs SSDS, P<0.99, t = 0.40, df = 12; Control vs CSDS, P<0.0017, t = 4.63, df = 12; Bonferroni’s adjustments. **C.** % Immobile time during TST Control, SSDS, and CSDS: Two-way ANOVA, P < 0.0005, F(2, 12) = 14.98; Control vs SSDS, P<0.65, t = 1.3, df = 12; Control vs CSDS, P<0.0006, t = 5.25, df = 12; Bonferroni’s adjustments. **D.** % Time in the center during OFT Control, SSDS, and CSDS: Two-way ANOVA, P < 0.017, F(2, 12) =5.86; Control vs SSDS, P<0.99, t = 0.33, df = 12; Control vs CSDS, P<0.03, t = 3.13, df = 12; Bonferroni’s adjustments. **E.** Total distance traveled during OFT Control, SSDS, and CSDS: Two-way ANOVA, P < 0.056, F(2, 12) = 3.71; Control vs SSDS, P<0.70, t = 1.25, df = 12; Control vs CSDS, P<0.056, t = 2.73, df = 12; Bonferroni’s adjustments. All data are shown as mean ± s.e.m.

**
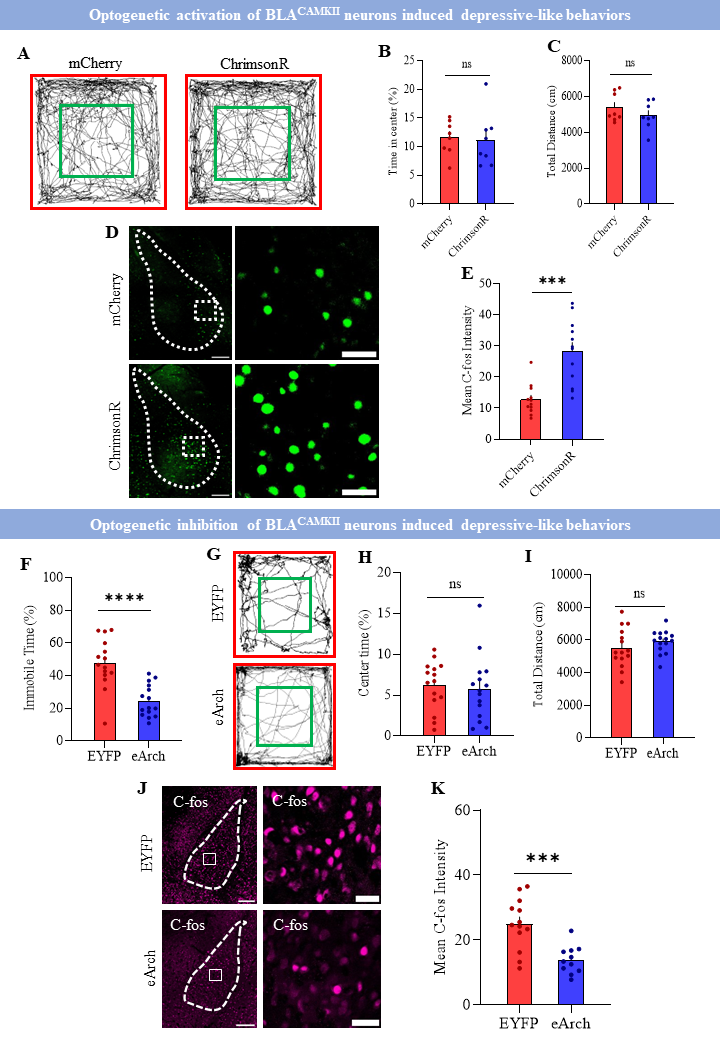
**

**Figure S3: BLA^CAMKII^ mediates depressive-like behaviors.**

**A.** Showing the trajectory map of mcherry and ChrimsonR in the OFT. **B.** % Time in the center during OFT mCherry vs ChrimsonR, P < 0.83, t = 0.224, df = 14, N = 8, two-tailed unpaired t-test. **C.** Total distance traveled during OFT mCherry vs ChrimsonR P < 0.28, t = 1.12, df = 14, N = 8, two-tailed unpaired t-test. **D.** Representative images showing the c-fos activity in the BLA of mCherry and ChrimsonR group. The scale bar was set to 200 um on the left images and zoomed on the right 50 um. **E.** Showing the mean c-fos intensity in the BLA of mCherry and ChrimsonR group, P < 0.0002, t = 4.66 df = 22, Slices n = 12 from N = 3 mice, two-tailed unpaired t-test. **F.** Immobile duration during the tail suspension test between EYFP vs eArch group, P < 0.0001, t = 4.94, df = 28, N = 15 mice, two-tailed unpaired t-test. **G.** Showing the trajectory map of EYFP vs eArch. **H.** % Center time in the OFT, P < 0.74, t = 0.33, df = 28, N = 15 mice, two-tailed unpaired t-test. **I.** Total distance (cm) traveled in OFT, P < 0.29, t = 1.1, df = 28, N = 15 mice, two-tailed unpaired t-test. **J.** Showing the c-fos images of EYFP and eArch group, Scale bar = 200 um (left), magnified images shown on right with scale bar = 50 um. **K.** Showing the mean c-fos intensity EYFP and eArch, P < 0.0004, t = 4.12, df = 22, n = 12 slices from N = 3 mice, two-tailed unpaired t-test. All data are shown as mean ± s.e.m.

**
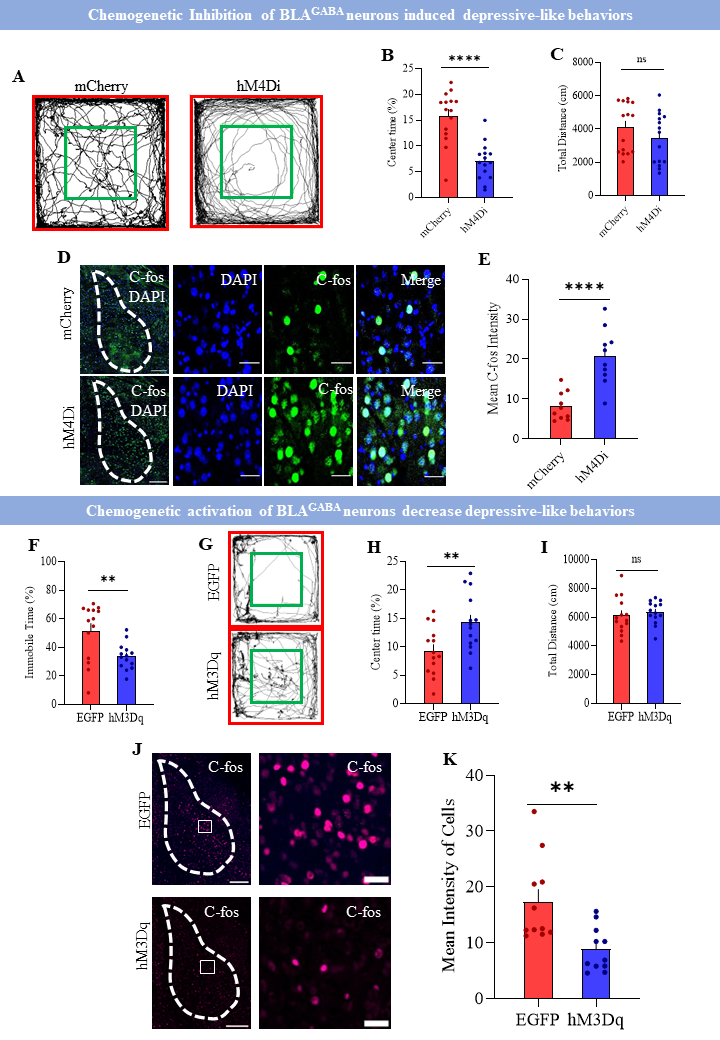
**

**Figure S4: BLA^GABA^ modulates depressive-like behaviors.**

**A.** Showing the trajectory map of mCherry and hM4Di. **B.** % Center time in the OFT, P < 0.0001, t = 5.57, df = 28, N = 15 mice, two-tailed unpaired t-test. **C.** Total distance (cm) traveled in OFT, P < 0.24, t = 1.19, df = 28, N = 15 mice, two-tailed unpaired t-test. **D.** Showing the c-fos images of mCherry and hM4Di group, Scale bar = 200 um (left), magnified images shown on right with scale bar = 50 um. **E.** Showing the mean c-fos intensity mCherry vs hM4Di, P < 0.0001, t = 5.044, df = 18, n = 10 slices from N = 3 mice, two-tailed unpaired t-test. **F.** Immobile duration during the tail suspension test between EGFP and hM3Dq, P < 0.0051, t = 3.058, df = 26, N = 14 mice, two-tailed unpaired t-test. **G.** Showing the trajectory map of EGFP and hM3Dq. **H.** % Center time in the OFT among EGFP and hM3Dq, P < 0.0093, t = 2.81, df = 26, N = 14 mice, two-tailed unpaired t-test. **I.** Total distance travel in OFT, P < 0.59, t = 0.55, df = 26, N = 14 mice, two-tailed unpaired t-test. **J.** Showing the c-fos images of EGFP and hM3Dq group, Scale bar = 200 um (left), magnified images shown on right with scale bar = 50 um. **K.** Showing the mean c-fos intensity EGFP and hM3Dq, P < 0.0035, t = 3.312, df = 20, n = 11 slices from N = 3 mice, two-tailed unpaired t-test. All data are shown as mean ± s.e.m.

**
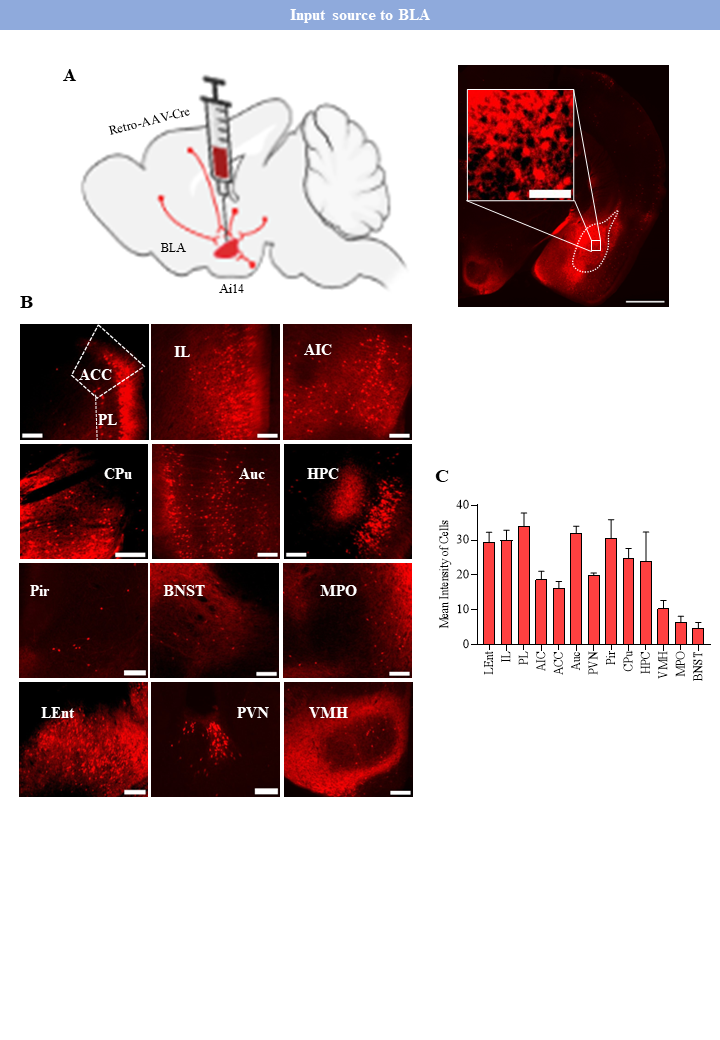
**

**Figure S5: Input source to BLA**

**A.** Left: Strategy for the tracing of synaptic input to the BLA using Retro-AAV-CRE, Right: virus injection site BLA and virus expression. **B.** Representative images showing the retrogradely labeled cells in different regions (anterior cingulate cortex (ACC), prelimbic cortex (PL), infralimbic cortex (IL), agranular insular cortex (AIC), piriform cortex (Pir), bed nucleus of stria terminal (BNST), medial preoptic nucleus (MPO), caudate putamen (CPu), Auditory cortex (Auc), Hippocampus (HPC), Lateral entorhinal cortex (LEnt), Paraventricular nucleus (PVN), ventromedial hypothalamus (VMH)), scale bar set to 100 um. **C.** Showing the mean intensity of cells in the BLA input region.

**Table S1: Key resources.**

| **REAGENT OR RESOURCES** | **SOURCE** | **IDENTIFIER** |
| --- | --- | --- |
| **Antibodies** | | |
| Anti-cfos (Rabbit polyclonal) | Abcam | Ab190289 |
| Anti-Rabbit IgG (Alexa Fluor 647) | Thermofishers scientific | Cat# A-21245 |
| Anti-Rabbit IgG (Alexa Fluor 488) | Thermofishers scientific | Cat# A-11008 |
| Anti-Rabbit IgG (Alexa Fluor 405) | Thermofishers scientific | Cat# A-31556 |
| **Bacterial and Virus strains** | | |
| AAV9-CAMKII-GCAMP6s | Addgene | Adgene_50459 |
| AAV9-hDlx-hM4Di-dtomato | Addgene | Cat# 83896 |
| AAV9-mCAMKII-DIO-ChrimsonR-mCherry | Taitool Bioscience | Cat# S0728-9 |
| AAV9-EF1a-DIO-EYFP | Brain VTA | Cat# PT-0012 |
| AAV9-mDlx-DIO-ChrimsonR-mCherry | Brain VTA | N/A |
| AAV9-mDlx-DIO-mCherry | Brain VTA | N/A |
| AAV9-mDlx-DIO-hM3Dq-EGFP | Brain VTA | Cat# PT-2654 |
| AAV9-mDlx-Cre | Brain VTA | Cat# PT-0306 |
| AAV9-CAMKII-Cre | Brain VTA | Cat# PT-0220 |
| Retro-AAV-hSyn-Cre | Addgene | Cat#105553 |
| AAV9-hSyn-DIO-EGFP | Taitool Bioscience | Cat#S0746-9 |
| AAV9-hSyn-DIO-eArchT3.0-EGFP | Taitool Bioscience | Cat# S0275-9 |
| **Chemicals, peptides, and recombinant proteins** | | |
| DAPI | Santa Cruz Biotechnology | Cat# sc-3598 |
| Pentobarbital sodium | Alfasan International B.V. | N/A |
| CNO | MedChem Express. | Cat# 17366 |
| **Experimental models: Organisms/strains** | | |
| Mouse: C57BL/6J | Laboratory Animal Research Unit, City University of Hong Kong. | N/A |
| Mouse: ICR Retired Breeder | Laboratory Animal Research Unit, City University of Hong Kong. | N/A |
|  | The Laboratory Animal Services Centre; Chinese University of Hong Kong. |  |
| Mouse: Ai14 reporter mice | Jackson Laboratory | Stock# 007914 |
| **Software and algorithms** | | |
| Fiji | Schindelin et al., 2012 | https://imagej.net/software/fiji/ |
| Smart 3.0 | Pan Lab | https://www.panlab.com/en/products/smart-video-tracking-software-panlab |
| GraphPad Prism 8.0.1 | GraphPad Prism | https://www.graphpad.com/scientific-software/prism/ |
| Synapse Suite | Tucker-Davis Technologies | <http://www.tdt.com/component/snapse-software/> |
| Debut Video Capture | NCH Software | <https://www.nch.com.au/general/sitemap.html> |
